# Structural heterogeneity and functional diversity of topologically associating domains in mammalian genomes

**DOI:** 10.1093/nar/gkv684

**Published:** 2015-07-06

**Authors:** Xiao-Tao Wang, Peng-Fei Dong, Hong-Yu Zhang, Cheng Peng

**Affiliations:** Agricultural Bioinformatics Key Laboratory of Hubei Province, College of Informatics, Huazhong Agricultural University, Wuhan 430070, China

## Abstract

Recent chromosome conformation capture (3C) derived techniques have revealed that topologically associating domain (TAD) is a pervasive element in chromatin three-dimensional (3D) organization. However, there is currently no parameter to quantitatively measure the structural characteristics of TADs, thus obscuring our understanding on the structural and functional differences among TADs. Based on our finding that there exist intrinsic chromatin interaction patterns in TADs, we define a theoretical parameter, called aggregation preference (AP), to characterize TAD structures by capturing the interaction aggregation degree. Applying this defined parameter to 11 Hi-C data sets generated by both traditional and *in situ* Hi-C experimental pipelines, our analyses reveal that heterogeneous structures exist among TADs, and this structural heterogeneity is significantly correlated to DNA sequences, epigenomic signals and gene expressions. Although TADs can be stable in genomic positions across cell lines, structural comparisons show that a considerable number of stable TADs undergo significantly structural rearrangements during cell changes. Moreover, the structural change of TAD is tightly associated with its transcription remodeling. Altogether, the theoretical parameter defined in this work provides a quantitative method to link structural characteristics and biological functions of TADs, and this linkage implies that chromatin interaction pattern has the potential to mark transcription activity in TADs.

## INTRODUCTION

Recent chromosome conformation capture (3C) derived techniques (1,2), especially Hi-C (3), have revealed that chromatins are partitioned into topologically associating domains (TADs), also called topological domains, in which the intra-domain chromatin interactions are significantly stronger than inter-domain interactions. Remarkably, TAD acts as a pervasive, or at least in part, structural element of chromatin three-dimensional (3D) organization across species, including *Caulobacter crescentus* (4), *Plasmodium falciparum* (5), Arabidopsis (6,7), yeast (8), drosophila (9), mouse (10,11) and human (11). Comparative analysis further shows that the genomic positions of TADs appear to be stable across cell types and conserved across species in mammals (11). Besides chromatin organization, TAD provides structural basis for chromatin regulation. It was found that most identified enhancer-promoter interactions were located in the same TADs (12). The study on mouse revealed that the limb development was regulated by the switch of two neighbor TADs (13). A recent work on bacteria *C. crescentus* provided the direct evidence that disrupting TADs leads to the change of gene expressions (4).

Given the importance of TADs, it is critical to interpret the structural characteristics of TADs and their association with biological functions. Previous works (4,9–11) annotated the TADs by integrating transcription factor (TF) binding and epigenomic signals, revealing that TADs could be different in biological functions. However, integrative annotation cannot reveal the structural characteristics of TADs. Thus, it is hard to build the linkage between structure and function in this way. To bridge the gap, the interaction frequency together with principle component analysis were previously used to identify open and close structures of chromatin regions at the genome-wide scale (3,6). Nevertheless, using this calculation to distinguish domain structures in the same class is difficult since specific chromatin interaction patterns are not considered. To our knowledge, there is currently no parameter to quantitatively measure the chromatin interaction patterns of TADs.

Inspired by our finding that TADs exhibit specific chromatin interaction patterns, we define a theoretical parameter, called aggregation preference (AP), to represent the structural characteristics of TAD and then build its association with biological functions. First, the locally high-frequency chromatin interactions violating distance-dependence decay are selected from TAD. Then a density-based algorithm DBSCAN (14) is used to cluster the selected chromatin interactions into groups according to their spatial aggregation. Finally, the weighted density of clustered groups is defined as AP to represent the interaction aggregation degree. This defined parameter is next used to investigate 11 chromatin interaction data sets on human and mouse cell types generated by traditional Hi-C and *in situ* Hi-C, a so-called improved pipeline published very recently (15). The Hi-C data on other species are excluded from this work because their domain sizes are too small at current resolution. Statistical analyses show that TADs are quite different in chromatin interaction patterns and these structural characteristics are significantly correlated to DNA sequence, epigenomic signals and transcription activity. Inter-cell-line comparison further reveals that the detailed structures of TADs can be significantly rearranged across cell lines although these domains are stable in genomic positions. Furthermore, the structural rearrangement is tightly associated with epigenomic remodeling and transcriptional regulation. Altogether, we define a theoretical parameter to build linkage between structural characteristics and biological functions of TADs.

## MATERIALS AND METHODS

### Hi-C data sources and processing

For traditional Hi-C experiments, the data sets of human cell line hESC and two mouse cell lines (mESC and Cortex) were downloaded from NCBI with accession number GSE35156 (11), and the data sets on human cell lines IMR90 and GM12878 were downloaded from NCBI with accession numbers GSE43070 (16) and GSE48592 (17), respectively. The *in situ* Hi-C data sets of human cell lines GM12878, IMR90, K562, HMEC, HUVEC and NHEK were downloaded from NCBI with accession number GSE63525 (15). Only the data sets generated by MboI restriction enzyme in the original work are used in this work, and the data sets on human (KBM7) and mouse (CH12-LX) cell lines are eliminated due to lack of epigenomic data.

The pipeline of Hi-C data processing follows a previous procedure (18) by using hg19 and mm10 as human and mouse reference genomes. Briefly, pair-end reads are mapped to the genome using an iterative mapping scheme, in which each read is truncated to 25 bp first and extended by 5 bp iteratively until the mapping with maximum read length is achieved. The uniquely mapped pair-end reads are used for the next analysis. To maintain data reliability, different types of noisy reads are removed from data sets, including the reads originated from polymerase-chain-reaction duplication, the reads started within 5 bp of the restriction site or located in the same restriction fragments, the reads located on very large (100 kb) or small (100 bp) fragments, the so-called random-break reads, and the reads that face towards each other and are separated by less than the library length. Fragments with the top 0.5% number of reads are also eliminated from data sets. The summary of Hi-C data is provided in Supplementary Table S1.

### Chromatin representation and TAD identification

Chromatin is partitioned into bins (or beads) at given resolution, and the kept reads are assigned to these beads to generate the observed interaction matrix $(O_{ij} )_{N \times N}$ for each Hi-C data set, where *N* is the number of beads in the chromatin and $O_{ij}$ is the observed interaction frequency between beads *i* and *j*. The gap regions arising from DNA repeat elements or the absence of enzyme restriction sites are eliminated from the observed interaction matrix to facilitate next calculations (19). To remove experimental biases, interaction matrix is corrected by using hiclib package (18) with default relative error (10%). The corrected matrix $(f_{ij} )_{N \times N}$ is used as input to perform TAD identification by using directionality index (DI) based hidden Markov model (HMM) (11).

### Chromatin interaction parameter calculation

The statistically significant chromatin interactions are selected by considering both distance-dependent decay and local interaction background (15). Specifically, the averaged interaction frequency at given genomic distance *k* in a *n*-beads TAD is calculated by $f(k) = \frac{1}{{n - k}}\sum\limits_{\left| {i - j} \right| = k} {f_{ij} }$. Then B-Spline is used to approximate the curve between $f(k)$ and *k* to obtain the smoothed interaction frequency $S(k)$. Generally, the smoothed curve will fluctuate after the first turning point $k_r$ due to the shortage of super long-range chromatin interactions in the TAD. To solve this problem, the averaged interaction frequency is finally set to $E_{i,j} = \left\{ {\begin{array}{*{20}c} {S(\left| {i - j} \right|),\;\;\left| {i - j} \right| \le k_r } \\ {S(k_r ),\;\;\left| {i - j} \right| >k_r } \\\end{array}} \right.$, where $(i,j)$ represents given chromatin interaction in the TAD. Besides genomic distance, the expected interaction frequency of chromatin interaction $(i,j)$ is calculated by using square windows to further take local interaction background into consideration: 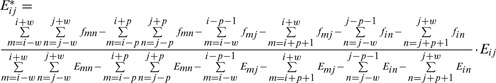 where $2p + 1$ and $2w + 1$ specify the widths of two square windows centered at $(i,j)$ with $p < w$. Then the *P*-value for observing interaction frequency $floor(f_{ij} )$ is calculated based on the Poisson process with expected value $\lambda _{ij} = E_{ij}^*$. To further reduce noisy chromatin interactions, the chromatin interactions with low windowed interaction frequency $C_{ij} = \sum\limits_{m = i - p}^{i + p} {\sum\limits_{n = j - p}^{j + p} {f_{mn} } }$ are eliminated from next interaction selection (around 30% in this work to account for low sequencing depths in some data sets). Then ∼5% of chromatin interactions with highest statistical significance in the TAD are selected for next analysis. By following previous work (15), the window parameters *p* and *w* are set to 4 and 7 respectively at 5 kb resolution, 2 and 5 at 10 kb resolution, and 1 and 3 at 20 kb resolution.

The selected chromatin interactions are clustered into spatially neighbored groups $G_i$, called interaction blocks in this work, by using the density-based algorithm DBSCAN (14). This algorithm utilizes the fact that the clustered points tend to have high local density and the isolated points tend to have low local density. In the calculation, the point density is defined to be the number of points located in the spatial sphere at a given radius. Two parameters, i.e. the minimum number of points and the spatial radius, are involved in this calculation. Previous work (20) has discussed how to set these two parameters, and our calculation follows this procedure.

The convex hull $H_i$ is calculated from interaction block $G_i$ by using QuickHull algorithm (21). The so-called core-point $p_j$ in DBSCAN is defined as the point surrounded by at least $n_p$ points (aforementioned minimum number of points) in convex hull $H_i$, and its local density is calculated by $dp_j = {{n_p }}\left/ {\sum\limits_{m = 1}^{n_p } {dist(p_j ,p_m )} } \right.$, where $p_m$ is the $m^{th}$ closest point to target point $p_j$, and $dist()$ denotes the squared distance between two points. The interaction density of convex hull $H_i$ is calculated by $d_i = \sum\limits_{p_j \in H_i } {dp_j } /cpt(H_i )$, where $cpt(H_i )$ is the number of core-points in the convex hull $H_i$. The density of isolated convex hull is set to zero. Finally, chromatin interaction aggregation preference of this domain is defined as the weighted density: $d = \sum\limits_i {\frac{{pts(H_i )}}{{pts(TAD)}}d_i }$, where $pts(H_i )$ and $pts(TAD)$ mean the numbers of selected interactions in the convex hull $H_i$ and TAD, respectively. It is worth noting that other parameters may also be developed to characterize TAD structures by using these significant interactions.

### TAD annotation and comparison

The DNA sequence features, such as transcription start sites (TSSs), short interspersed nuclear elements (SINEs) and long interspersed nuclear elements (LINEs), were downloaded from ENCODE (22). LaminB1-associated binding signals in mESC were obtained from the reference (23), in which the nucleotides have already been processed to be binding or unbinding ones. The ChIP-Seq, including TF binding and epigenomic signals, and processed RNA-Seq reads were downloaded from ENCODE (22). The ChIP-Seq signals were uniformly normalized by using the align2rawsignal software (A. Kundaje, http://code.google.com/p/align2rawsignal/). When calculating Pearson correlation between AP and given annotation signal, the processed signal in the domain are summed and then averaged by domain length to represent signal strength of TAD. To reduce the negative impact of confounding factors, such as GC-composition of the genome, in computing *P*-value for correlation coefficient, the Pearson correlation between AP and randomly shuffled annotation signal (less than 1 Mb independently for different chromosomes) is calculated. This shuffled correlation is repeated 500 times independently, and the final *P*-value is computed by testing the difference between original correlation coefficient and shuffled correlation average under Fisher's z transformation (24).

TAD stability is measured by using the boundary overlap in genomic positions between two cell lines or between two experimental pipelines on the same cell line. The number of stable TADs decreases as stricter criterion is used (Supplementary Figure S1). To compromise between TAD number and stability, TADs sharing similar boundaries (less than 5 bins in both left and right ones) in genomic positions are considered to be stable. The overlapped block regions of two stable TADs are divided by total blocks in each cell line, and the averaged overlap percentage is used to calculate Pearson correlation between AP change and block overlap. By using the biological replicates sharing similar intra-chromosomal interaction reads in different cell lines, statistical analysis shows that the stable TADs consistently exhibit higher structural variations arising from cell lines than those arising from biological replicates (Supplementary Figure S2). To simplify the TAD selection when performing structural comparisons across cell lines in diverse cases, quantile regression is used to select stable TADs with top AP change by accounting for the difference in structural variations (25,26). Finally, around 20% of stable TADs are selected to perform structural comparisons in detail (Supplementary Figure S3). When investigating the association between parameter AP and annotation signals in TADs, the Wilcoxon signed rank test is used to calculate the *P*-value for each investigated signal.

## RESULTS

### Parameter calculation from Hi-C chromatin interactions

Five traditional Hi-C maps are denoted as hESC-T, GM12878-T, IMR90-T, mESC-T and Cortex-T to represent three human cell lines and two mouse cell lines, while six *in situ* Hi-C maps are denoted as GM12878-I, IMR90-I, K562-I, HMEC-I, HUVEC-I and NHEK-I to represent corresponding human cell lines. To compromise between higher resolution and limited sequencing depth, TADs were identified at 20 kb resolution for traditional Hi-C data sets and at 10 kb resolution for *in situ* Hi-C data sets by using DI based HMM proposed by a previously work (11). For three deepest sequencing data sets (GM12878-I, IMR90-I and K562-I), additional TAD identifications were performed at 5 kb resolution. The validity of TAD identification at given resolutions has already been evaluated in both traditional and *in situ* Hi-C data sets elsewhere (11,15). Finally thousands of TADs were generated in each cell line (Supplementary Figure S4). Since different kinds of structural characteristics can be obtained at different resolutions due to hierarchical chromatin architecture (27), the TADs identified from different resolutions are not directly compared in this work.

**Figure 1.**
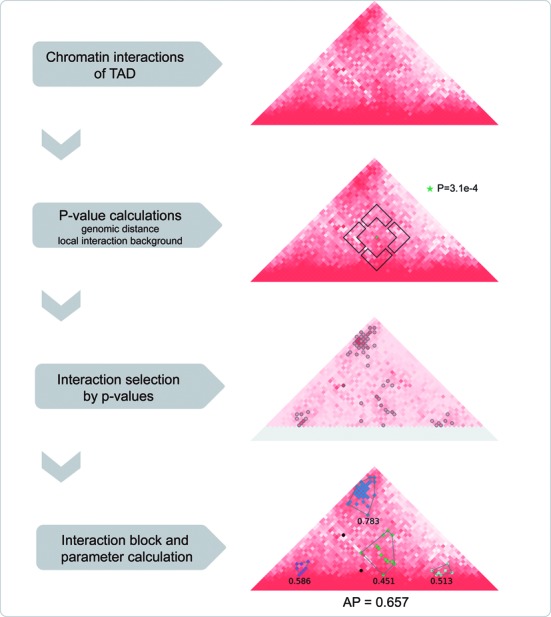
The computational pipeline of parameter AP. The first triangle represent the interaction heat map colored by interaction frequency. For given point (star) in the second heat map, square windows show the local interaction background when calculating the *P*-value (3.1e-4). The third heat map is colored by calculated *P*-values, in which circles are the selected significant interactions and the points in gray color are eliminated from interaction selection because of window sizes. In the bottom heat map, convex hulls denote the clustered chromatin interactions, i.e. interaction blocks, and corresponding individual densities. Black points are the isolated blocks.

Thorough inspection revealed the nonrandom distribution of those high-frequency chromatin interactions, which tend to form different types of spatial clusters (Figure 1). Inspired by this finding, we defined a novel parameter, called aggregation preference (AP), to represent the structural characteristics of TADs by calculating the aggregation degree of high-frequency chromatin interactions. First, the statistical significance of observing each chromatin interaction was calculated by considering both the genomic distance and local interaction background. Second, the chromatin interactions were sorted by calculated *P*-values, and ∼5% of top significant ones with locally high interaction frequencies were selected for next analysis. Third, the selected chromatin interactions were clustered into spatial groups, called interaction blocks in this work, by using a density-based algorithm DBSCAN (14). Finally, the parameter AP was defined as the weighted density of interaction blocks in each TAD (Materials and Methods). It can be expected that the parameter AP defined from selected interactions captures the structural characteristics of TAD based on the fluorescence *in situ* hybridization (FISH) observations that statistically significant interactions are often physically preferred contacts (6,8–10,12,15,28), despite essential difference between these two concepts due to the complicated nature of chromatin interactions (29).

Pearson correlations between biological replicates show that the defined parameter AP is highly reproducible in both traditional and *in situ* Hi-C data sets (Supplementary Figure S5). The cell lines hESC-T, HUVEC-I and NHEK-I are eliminated from reproducibility evaluation, either because significant difference exists in sequencing depth between two replicates or because there are no biological replicates. AP values calculated from merged replicates are used for next analysis in all cell lines. We next performed TAD identification and AP calculation for GM12878-I and IMR90-I at 20 kb resolution to further evaluate the robustness of parameter AP between two experimental pipelines in the same human cell lines. To reduce the impact of differences in sequencing depth and data quality, only the stable TADs shared by both Hi-C pipelines were used to calculate the reproducibility of parameter AP. Our result shows that the parameter AP is quite reproducible between two experimental pipelines (Supplementary Figure S6). Finally, these two kinds of reproducibility analyses together indicate the robustness of our defined parameter.

### Structural heterogeneity and functional implications of TADs

We explored the structural characteristics of TADs by using interaction blocks and corresponding parameter AP. Meanwhile, domain structure was functionally annotated by using DNA sequences, epigenomic signals and transcription activity. Among these signals, SINEs are often found in gene-rich regions (30) and LINEs are generally located in heterochromatin (31). Lamina-associated bindings are generally heterochromatin signals. H3K4me3 and H3K4me1 are promoter and enhancer markers, respectively. H3K27ac generally reflects that the enhancer and promoter are active or not. RNAPII binding and H3K36me3 reflect the transcription activity, and RNA sequence (RNASeq) directly represents transcription level (32).

**Figure 2.**
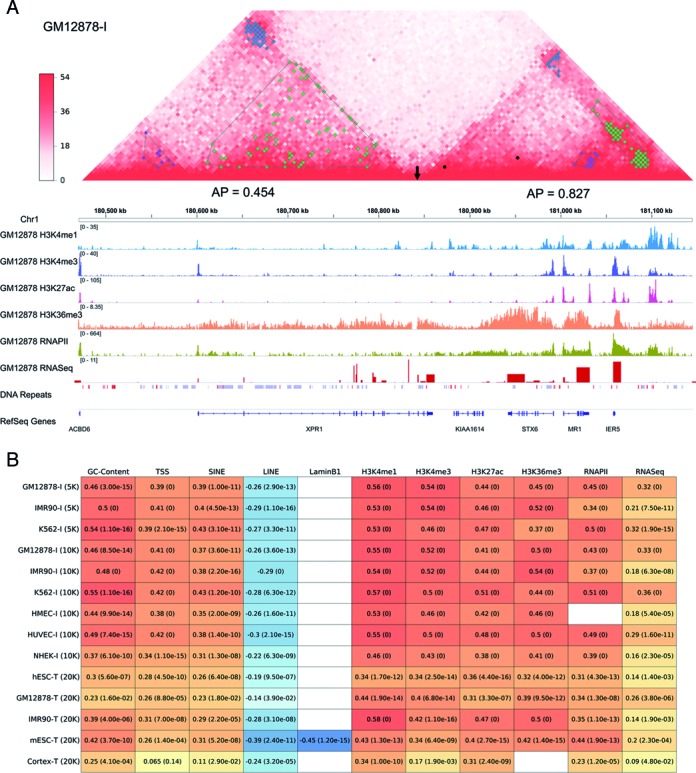
Structural characteristics and biological functions of TADs. (**A**) Structures and functions of two TADs from chromatin region (Chr1:180,470,000-181,145,000) in cell line GM12878-I at 5 kb resolution. The two-dimensional interaction heat map, epigenomic signals, RNA expression, DNA repeats and reference genes are shown. Arrow indicates the boundary position between two TADs. In DNA repeats, red and blue colors denote SINEs and LINEs, respectively. (**B**) Pearson correlation coefficients between AP value and annotation signals. Blank entries indicate the absence of annotation signals. *P*-values are shown in the brackets.

The interaction blocks generated from selected chromatin interactions are the structural and functional bases to distinguish TADs. Though previous works (16,33) have identified functional chromatin interactions on the same cell lines, our interaction selection is different from those works in terms of scientific purpose. Moreover, our method further utilizes the clustering patterns of locally high-frequency chromatin interactions to distinguish TADs. Figure 2A illustrates that TADs exhibit different types of interaction blocks. The chromatin interactions are dispersedly distributed to form low-density interaction blocks in the left domain, but the selected chromatin interactions are greatly aggregated to form high-density interaction blocks in the right domain. This structural difference is well captured by the defined parameter AP. The annotation signals show that the domain with high-density blocks is more enriched in active signals, such as SINEs, active epigenomic signals and RNA expression, implying that the structural characteristics of TAD is correlated to biological function. The same situation can also be observed in traditional Hi-C data sets (Supplementary Figure S7).

Statistical analysis was performed to systematically investigate the relationship between structure and function in TADs. The wide distributions of AP values suggest that TAD structures are heterogeneous in all cell lines (Supplementary Figure S8). Some domains have high AP values, indicating that the selected chromatin interactions are aggregated to form high-density and small-size interaction blocks. By contrast, the domains exhibiting low AP values own the dispersedly distributed chromatin interactions. Nevertheless, the widely distributed values suggest the mixed situations in most domains. Consistent with Figure 2A, the parameter AP also shows significant correlation to active signals and anti-correlation to inactive signals (LINE and LaminB1-association binding) in both traditional and *in situ* Hi-C data sets (Figure 2B). These results suggest that TADs with highly aggregated interactions are generally located in active regions but TADs with disperse interactions are generally located in inactive regions. However, most domains contain both active and inactive regions with diverse biological functions.

### Structural rearrangements of TADs across cell lines

We next investigated the structural rearrangements of TADs across cell lines by using the parameter AP. Our analysis showed that TADs could be stable in genomic positions across cell lines, consistent with previous study (11). We then calculated the Pearson correlation between AP change and block overlap on these stable TADs, obtaining considerably negative coefficients (Supplementary Table S2). This result shows that AP change can indicate the overlap degree of interaction blocks between stable TADs, but the relationship is a little complicated. Therefore, quantile regression was used to select different types of stable TADs with significant AP change to perform structural comparisons in detail (Supplementary Figure S3).

**Figure 3.**
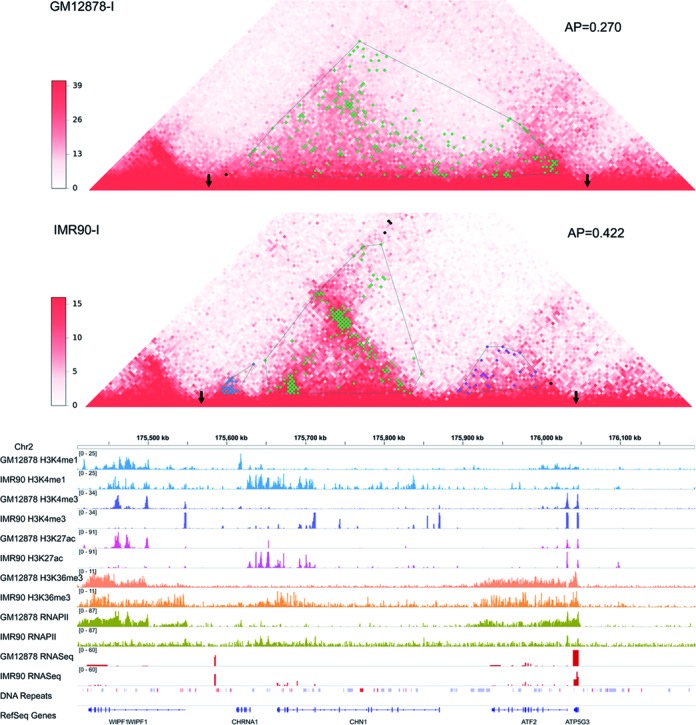
Structural rearrangement across human cell lines GM12878-I and IMR90-I. The TAD region and bidirectional extensions (Chr2: 175,410,000-176,195,000) are shown, including interaction heat maps at 5 kb resolution, clustered interaction blocks, epigeomic signals, RNA expression, DNA repeats and reference genes. The presentation scheme is the same as Figure 2A.

There exist different kinds of structural changes across cell lines, which can be captured by interaction blocks and corresponding AP values. Figure 3 illustrates that TAD can undergo block split when comparing human cell lines GM12878-I with IMR90-I. Intuitively, the statistically significant chromatin interactions in cell line GM12878-I are more dispersedly distributed compared with those in cell line IMR90-I, which is accurately captured by interaction blocks and the parameter AP. Together with aforementioned functional annotation, the structural change indicates that this TAD is activated in cell line IMR90. Consistently, the increase of active epigenomic and transcription signals (H3K4me1, H3K4me3, H3K27ac, H3K36mes, RNAPII and RNASeq) validates the increased transcription activity in cell line IMR90-I (Figure 3). In addition, the reverse direction of structural change, block merging, is also captured by our defined parameter (Supplementary Figure S9). Generally, the structural rearrangements of TADs are rather complicated, such as concurrent appearance, disappearance, split and merging of interaction blocks (Supplementary Figure S10), and thus the relationship between individual block change and functional change should be complex. However, the parameter AP can capture the overall structural and functional changes in various cases. The similar situations can be observed in traditional Hi-C data sets (Supplementary Figures S11 and S12).

**Figure 4.**
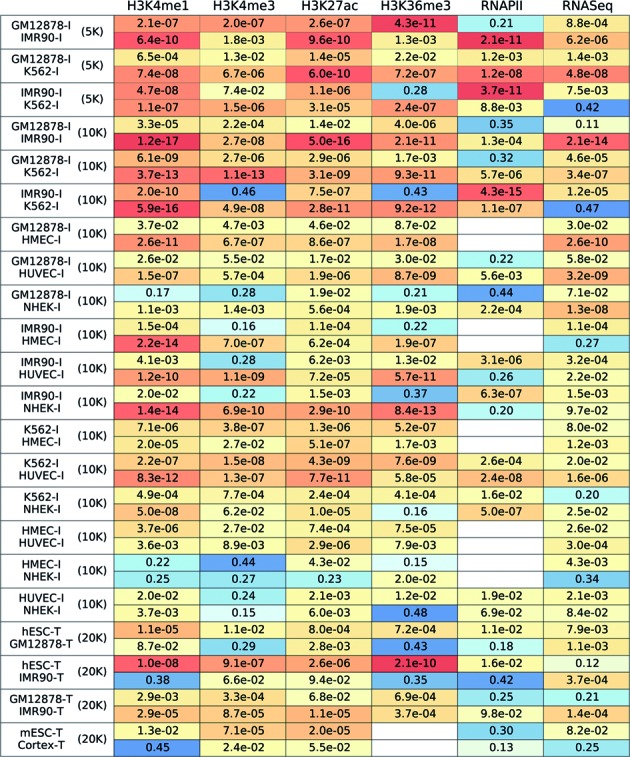
Statistical analysis on the association between structural rearrangement and functional signals. In each two-cell-line comparison, the participated cell lines and corresponding Hi-C resolution are shown in first column, and *P*-values in the first and second rows were calculated from selected TADs colored in orange and blue respectively in Supplementary Figure S3.

Genome-wide comparisons on the parameter AP revealed that a considerable number of TADs undergo structural changes in both human and mouse cell lines (Supplementary Figure S3). Some domains become more aggregated in chromatin interactions, and some domains transit to disperse chromatin interaction patterns. To statistically analyze the association between structural change and transcription remodeling, the selected TADs were paired by AP values, and Wilcoxon signed rank test was performed on epigenomic and transcription signals (Materials and Methods). Figure 4 shows that structural change is significantly associated with the remodeling of transcription activity in both traditional and *in situ* Hi-C cell lines, indicating that most TADs becoming interaction-aggregated are activated and those becoming interaction-dispersed are repressed in regulatory activity. The observed exceptions are partly caused by the complicated nature of structural variations and biological functions of TADs. These results suggest that chromatin interaction pattern captured by parameter AP has the potential to mark regulatory functions at genome-wide scale in mammalian genomes.

## DISCUSSION

Previous methods only focused on identifying functional chromatin interactions based on different purposes (16,33), but neglected the aggregation patterns of these chromatin interactions. In this paper, we utilize the intrinsic chromatin interaction pattern of TAD to define a novel parameter. By using this chromatin interaction parameter, we systematically investigate the structural characteristics of TADs and its association with biological functions in human and mouse cell lines, thus providing insights into chromatin 3D structure and functions in mammalian genomes.

Heterogeneous structures exist among TADs and this structural heterogeneity is probably encoded by DNA sequences and corresponding regulatory activities. Previous studies have shown that TAD is a pervasive element in chromatin 3D organization (4–11), but the structural difference among domains is not well understood. Statistical analysis on the defined feature reveals that the aggregation degree of chromatin interactions can be widely distributed among TADs, indicating the existence of diverse domain structures at high resolution. Integrative analysis further shows that euchromatin TADs have statistically higher chromatin interaction aggregation than heterochromatin TADs. This result can be partly explained by the fact that euchromatin regions are rich in genes and regulatory elements, which provide greater potential to form promoter-enhancer interaction clusters (34). Thus, the DNA sequence and regulatory activity may have resulted in the structural heterogeneity and functional diversity of TADs.

Chromatin interaction pattern has the potential to mark gene regulation and transcription activity, at least in TADs. Several recent works explored the relationship between chromatin interactions and biological functions. McCord et al. showed that the progress of Hutchinson–Gilford progeria syndrome was related to the reorganization of chromatin compartments (35). Rousseau et al. showed that chromatin conformation data in HOXA locus could be used to classify leukemia types, with better performance than RNA-Seq data (36). By combining polymer model with RNA-FISH, Giorgetti et al. revealed that transcription activities were coupled with chromatin conformation fluctuations in X-inactivation center (28). In this work, our systematical comparisons further show that structural change of TAD is significantly associated with its transcription remodeling in genome-wide scale. These results imply that chromatin interaction pattern has the potential to mark biological functions in mammalian genomes.

In summary, we define a parameter, the aggregation preference of statistically significant chromatin interactions, to represent the structural characteristics of TADs in this work, allowing us to systematically investigate domain structures and their association with biological functions. Statistical analysis shows that TADs are different in chromatin 3D structure, and this structural difference may be encoded in DNA sequences and corresponding regulatory activities. TADs can also undergo significant structural rearrangements across cell lines though they may preserve the domain positions in the genome. Moreover, this structural change is tightly associated with transcription remodeling. With the rapid expansion of Hi-C data, the chromatin interaction parameter defined in this work can provide a useful tool to explore the biological functions underlying the structural characteristics of TADs, especially when the epigenomic and TF binding signals are poor.

## AVAILABILITY

A TAD library (TADLib) to automatically calculate the parameter is coded by Python and freely available at https://pypi.python.org/pypi/TADLib.

## Supplementary Material

SUPPLEMENTARY DATA

## References

[1] Dekker J., Rippe K., Dekker M., Kleckner N. (2002). Capturing chromosome conformation. Science.

[2] Dostie J., Richmond T.A., Arnaout R.A., Selzer R.R., Lee W.L., Honan T.A., Rubio E.D., Krumm A., Lamb J., Nusbaum C. (2006). Chromosome Conformation Capture Carbon Copy (5C): a massively parallel solution for mapping interactions between genomic elements. Genome Res..

[3] Lieberman-Aiden E., van Berkum N.L., Williams L., Imakaev M., Ragoczy T., Telling A., Amit I., Lajoie B.R., Sabo P.J., Dorschner M.O. (2009). Comprehensive mapping of long-range interactions reveals folding principles of the human genome. Science.

[4] Le T.B., Imakaev M.V., Mirny L.A., Laub M.T. (2013). High-resolution mapping of the spatial organization of a bacterial chromosome. Science.

[5] Ay F., Bunnik E.M., Varoquaux N., Bol S.M., Prudhomme J., Vert J.P., Noble W.S., Le Roch K.G. (2014). Three-dimensional modeling of the P. falciparum genome during the erythrocytic cycle reveals a strong connection between genome architecture and gene expression. Genome Res..

[6] Grob S., Schmid M.W., Grossniklaus U. (2014). Hi-C analysis in Arabidopsis identifies the KNOT, a structure with similarities to the flamenco locus of Drosophila. Molecular cell.

[7] Wang C., Liu C., Roqueiro D., Grimm D., Schwab R., Becker C., Lanz C., Weigel D. (2015). Genome-wide analysis of local chromatin packing in Arabidopsis thaliana. Genome Res..

[8] Mizuguchi T., Fudenberg G., Mehta S., Belton J.M., Taneja N., Folco H.D., FitzGerald P., Dekker J., Mirny L., Barrowman J. (2014). Cohesin-dependent globules and heterochromatin shape 3D genome architecture in S. pombe. Nature.

[9] Sexton T., Yaffe E., Kenigsberg E., Bantignies F., Leblanc B., Hoichman M., Parrinello H., Tanay A., Cavalli G. (2012). Three-dimensional folding and functional organization principles of the Drosophila genome. Cell.

[10] Nora E.P., Lajoie B.R., Schulz E.G., Giorgetti L., Okamoto I., Servant N., Piolot T., van Berkum N.L., Meisig J., Sedat J. (2012). Spatial partitioning of the regulatory landscape of the X-inactivation centre. Nature.

[11] Dixon J.R., Selvaraj S., Yue F., Kim A., Li Y., Shen Y., Hu M., Liu J.S., Ren B. (2012). Topological domains in mammalian genomes identified by analysis of chromatin interactions. Nature.

[12] Phillips-Cremins J.E., Sauria M.E., Sanyal A., Gerasimova T.I., Lajoie B.R., Bell J.S., Ong C.T., Hookway T.A., Guo C., Sun Y. (2013). Architectural protein subclasses shape 3D organization of genomes during lineage commitment. Cell.

[13] Andrey G., Montavon T., Mascrez B., Gonzalez F., Noordermeer D., Leleu M., Trono D., Spitz F., Duboule D. (2013). A switch between topological domains underlies HoxD genes collinearity in mouse limbs. Science.

[14] Ester J., Kriegel H.P., Sander J., Xu J. (1996). A density-based algorithm for discovering clusters in large spatial databases with noise. Proc. 2nd Int. Conf. Knowledge Discovery and data mining (KDD’96).

[15] Rao S.S., Huntley M.H., Durand N.C., Stamenova E.K., Bochkov I.D., Robinson J.T., Sanborn A.L., Machol I., Omer A.D., Lander E.S. (2014). A 3D map of the human genome at kilobase resolution reveals principles of chromatin looping. Cell.

[16] Jin F., Li Y., Dixon J.R., Selvaraj S., Ye Z., Lee A.Y., Yen C.A., Schmitt A.D., Espinoza C.A., Ren B. (2013). A high-resolution map of the three-dimensional chromatin interactome in human cells. Nature.

[17] Selvaraj S., J R.D., Bansal V., Ren B. (2013). Whole-genome haplotype reconstruction using proximity-ligation and shotgun sequencing. Nature biotechnology.

[18] Imakaev M., Fudenberg G., McCord R.P., Naumova N., Goloborodko A., Lajoie B.R., Dekker J., Mirny L.A. (2012). Iterative correction of Hi-C data reveals hallmarks of chromosome organization. Nature methods.

[19] Peng C., Fu L.Y., Dong P.F., Deng Z.L., Li J.X., Wang X.T., Zhang H.Y. (2013). The sequencing bias relaxed characteristics of Hi-C derived data and implications for chromatin 3D modeling. Nucleic Acids Res.

[20] Daszykowski M., Walczak B., Massart D.L. (2002). Looking for natural patterns in analytical data. 2. Tracing local density with OPTICS. J. Chem. Inf. Comput. Sci..

[21] Barber C.B., Dobkin D.P., Huhdanpaa H.T. (1996). The Quickhull algorithm for convex hulls. ACM Trans. Math. Software.

[22] Bernstein B.E., Birney E., Dunham I., Green E.D., Gunter C., Snyder M. (2012). An integrated encyclopedia of DNA elements in the human genome. Nature.

[23] Peric-Hupkes D., Meuleman W., Pagie L., Bruggeman S.W., Solovei I., Brugman W., Graf S., Flicek P., Kerkhoven R.M., van Lohuizen M. (2010). Molecular maps of the reorganization of genome-nuclear lamina interactions during differentiation. Molecular cell.

[24] Fisher R.A. (1921). On the ‘Probable Error’ of a Coefficient of Correlation deduced from a Small Sample. Metron.

[25] Bolstad B.M., Irizarry R.A., Astrand M., Speed T.P. (2003). A comparison of normalization methods for high density oligonucleotide array data based on variance and bias. Bioinformatics.

[26] Bardet A.F., He Q., Zeitlinger J., Stark A. (2012). A computational pipeline for comparative ChIP-seq analyses. Nature protocols.

[27] Sexton T., Cavalli G. (2015). The role of chromosome domains in shaping the functional genome. Cell.

[28] Giorgetti L., Galupa R., Nora E.P., Piolot T., Lam F., Dekker J., Tiana G., Heard E. (2014). Predictive polymer modeling reveals coupled fluctuations in chromosome conformation and transcription. Cell.

[29] Dekker J., Marti-Renom M.A., Mirny L.A. (2013). Exploring the three-dimensional organization of genomes: interpreting chromatin interaction data. Nature reviews. Genetics.

[30] Batzer M.A., Deininger P.L. (2002). Alu repeats and human genomic diversity. Nature reviews Genetics.

[31] Grewal S.I., Jia S. (2007). Heterochromatin revisited. Nature reviews Genetics.

[32] Bickmore W.A., van Steensel B. (2013). Genome architecture: domain organization of interphase chromosomes. Cell.

[33] Ay F., Bailey T.L., Noble W.S. (2014). Statistical confidence estimation for Hi-C data reveals regulatory chromatin contacts. Genome Res..

[34] de Laat W., Duboule D. (2013). Topology of mammalian developmental enhancers and their regulatory landscapes. Nature.

[35] McCord R.P., Nazario-Toole A., Zhang H., Chines P.S., Zhan Y., Erdos M.R., Collins F.S., Dekker J., Cao K. (2013). Correlated alterations in genome organization, histone methylation, and DNA-lamin A/C interactions in Hutchinson-Gilford progeria syndrome. Genome Res..

[36] Rousseau M., Ferraiuolo M.A., Crutchley J.L., Wang X.Q., Miura H., Blanchette M., Dostie J. (2014). Classifying leukemia types with chromatin conformation data. Genome biology.

