# Supplementary material for: Structural heterogeneity and functional diversity of topologically associating domains in mammalian genomes
Source: Nucleic Acids Res. 2015 Jul 6;43(15):7237–46. doi: 10.1093/nar/gkv684 (PMC4551926; doi:10.1093/nar/gkv684)
Supplement: SUPPLEMENTARY DATA [file supp_43_15_7237__index.html]

Structural heterogeneity and functional diversity of topologically associating domains in mammalian genomes — Structural heterogeneity and functional diversity of topologically associating domains in mammalian genomes — SUPPLEMENTARY DATA 

# Structural heterogeneity and functional diversity of topologically associating domains in mammalian genomes

## SUPPLEMENTARY DATA

- SUPPLEMENTARY DATA
