# Supplementary material for: Structural heterogeneity and functional diversity of topologically associating domains in mammalian genomes
Source: Nucleic Acids Res. 2015 Jul 6;43(15):7237–46. doi: 10.1093/nar/gkv684 (PMC4551926; doi:10.1093/nar/gkv684)
Supplement: SUPPLEMENTARY DATA [file supp_gkv684_nar-03461-n-2014-File006.pdf]

SUPPLEMENTARY DATA

Structural heterogeneity and functional diversity of topologically associating domains in mammalian genomes

Xiao-Tao Wang, Peng-Fei Dong, Hong-Yu Zhang, Cheng Peng*

Agricultural Bioinformatics Key Laboratory of Hubei Province, College of Informatics, Huazhong Agricultural University, Wuhan 430070, China.

*To whom correspondence should be addressed.
Email: pengcheng@mail.hzau.edu.cn

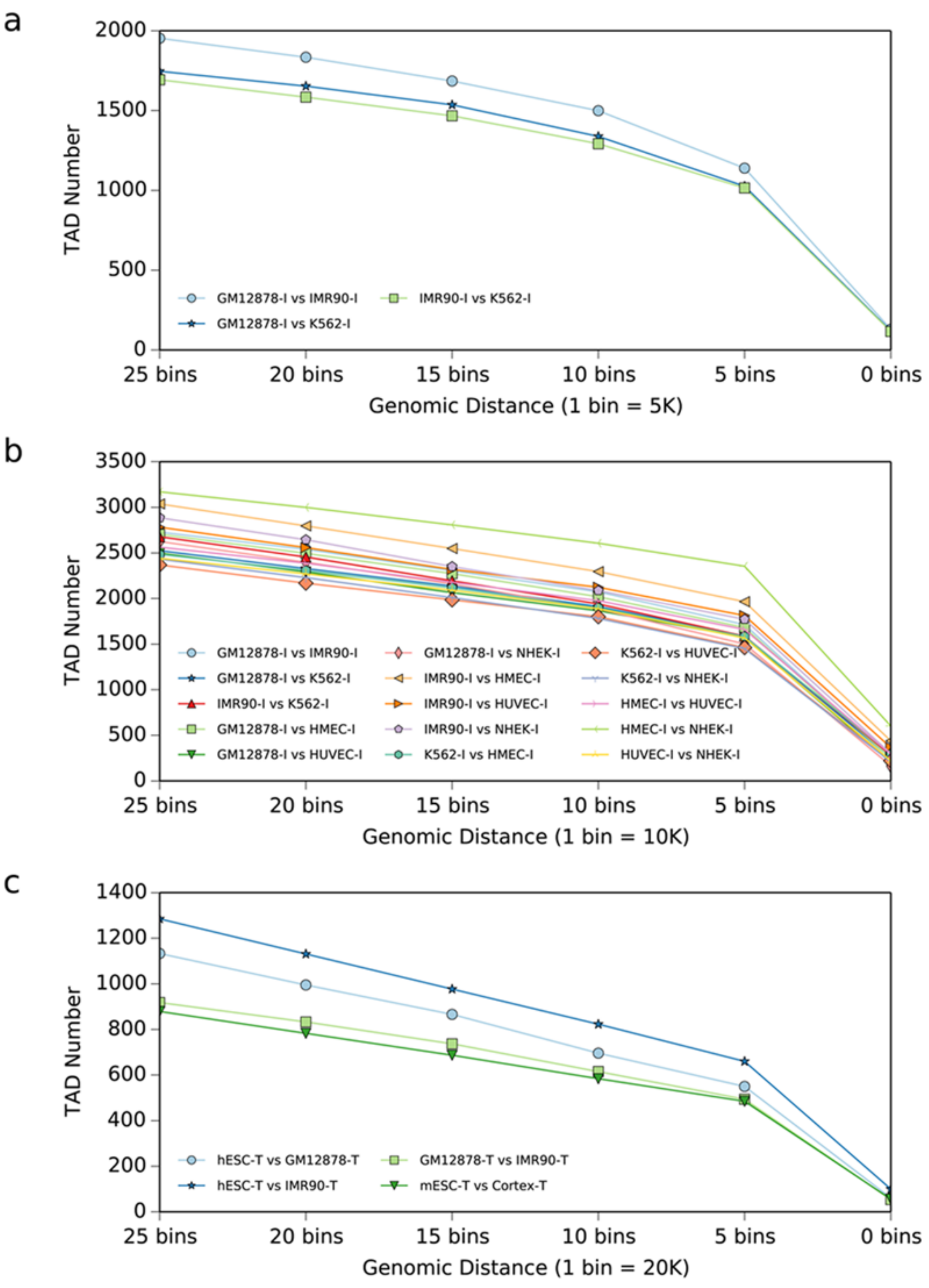

Supplementary figure 1. Statistics on position stability of TADs across cell lines. The x-axis means that both the left and right boundaries of two TADs are less than given genomic distance, and y-axis indicates the number of TADs sharing boundaries in two cell lines. (a) TAD stability at 5 kb resolution. (b) TAD stability at 10 kb resolution. (c) TAD stability at 20 kb resolution.

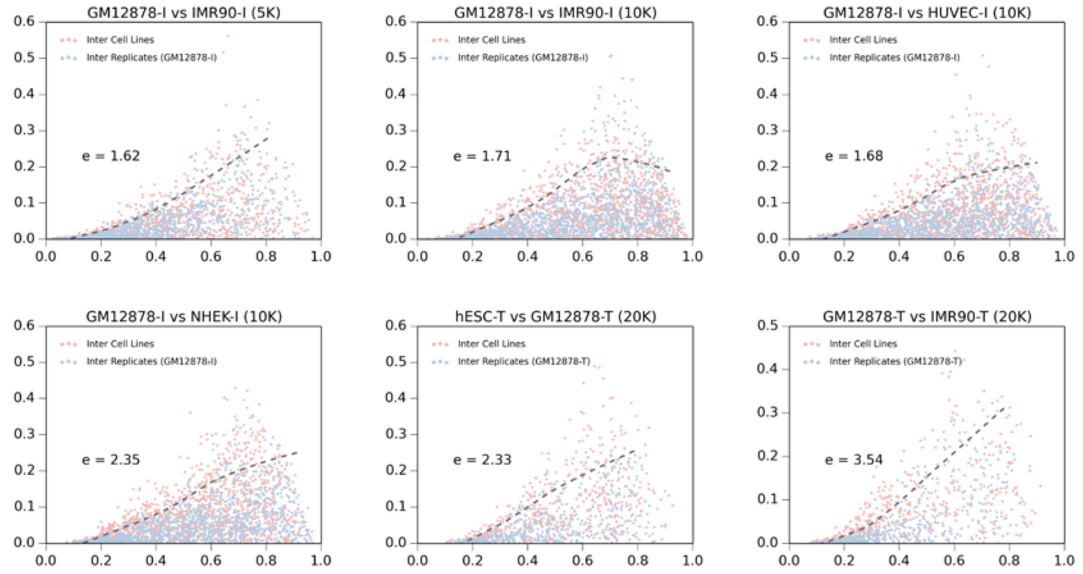

Supplementary figure 2. Statistical comparison between inter cell-line structural variations and inter replicate structural variations. Similar to Bland-Altman plot, x-axis and y-axis represent the pair $((AP_1 + AP_2)/2, |AP_1 - AP_2|)$, where AP_1 and AP_2 are two AP values calculated from two replicates (blue point) or two cell lines (red point). Each point denotes a TAD in comparison. The dash gray line shows the three-fold local standard deviation of absolute difference $|AP_1 - AP_2|$ from two inter replicates. The enrichment (e) is calculated as the number of red points divided by the number of blue points located above the dash gray line. The consistent enrichments (e>1) show that the structural variations between cell types are generally higher than those between biological replicates. To perform fair comparison, only the biological replicates with similar intra-chromosomal reads in different cell lines are selected for statistical comparison. In detail, the IMR90-I-R2, HUVEC-I, NHEK-I, GM12878-I-R5 and GM12878-I-R7 are selected for *in situ* Hi-C datasets, and hESC-T-R2, IMR90-T-R2, GM12878-T-R1 and GM12878-T-R2 are selected for traditional Hi-C datasets (Supplementary table 1).

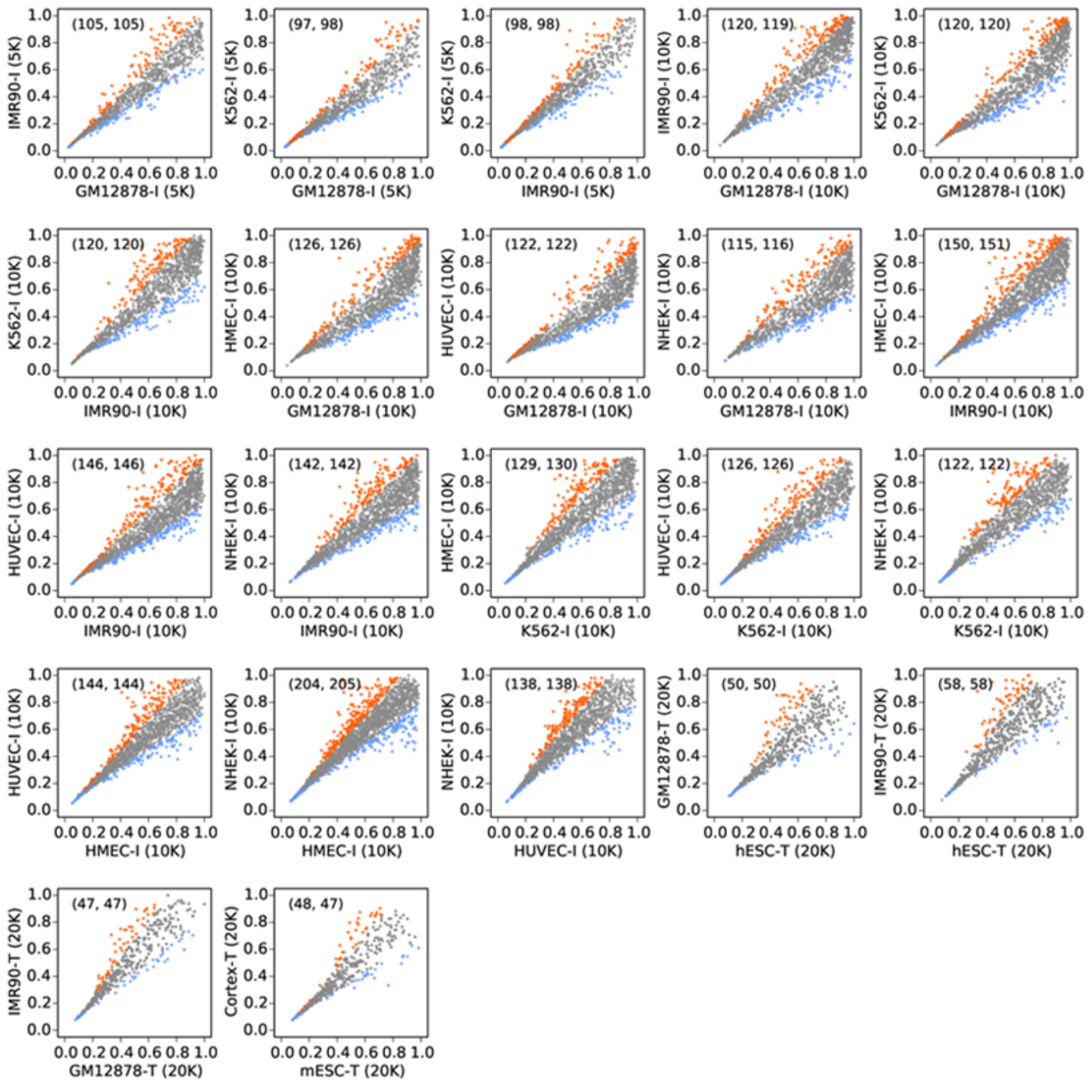

Supplementary figure 3. TAD selection across cell lines. In each subplot, the points represent the stable TADs with boundary overlap in two cell lines, and x-axis and y-axis denote AP values in corresponding cell lines. Orange and blue points represent the selected TADs for statistical association analysis in Figure 4, and the corresponding number pairs are shown in brackets.

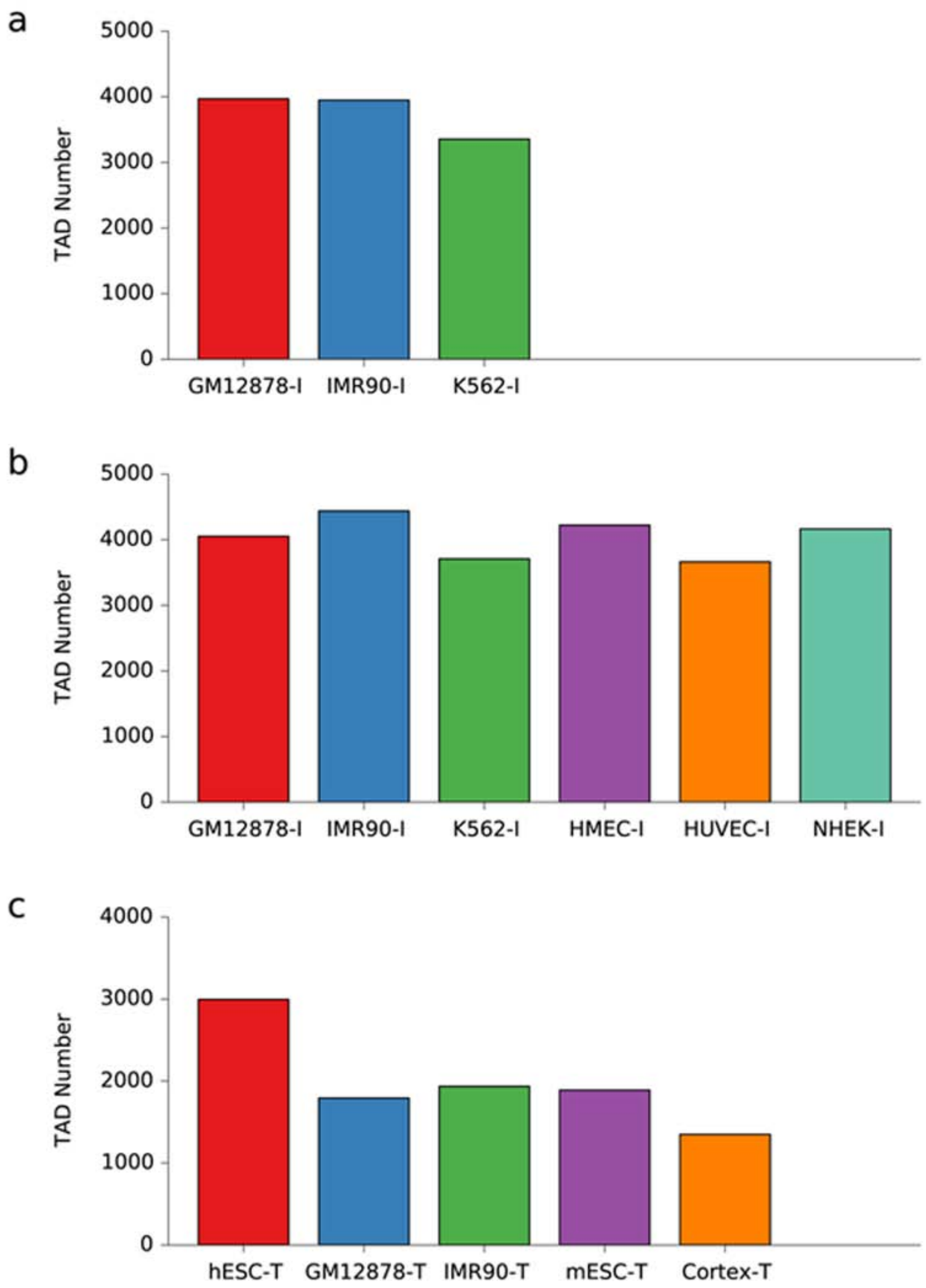

Supplementary figure 4. The numbers of identified TADs in different cases. (a) TAD numbers of three deepest sequencing datasets at 5 kb resolution. (b) TAD numbers of *in situ* Hi-C datasets at 10 kb resolution. (c) TAD numbers of traditional Hi-C datasets at 20 kb resolution.

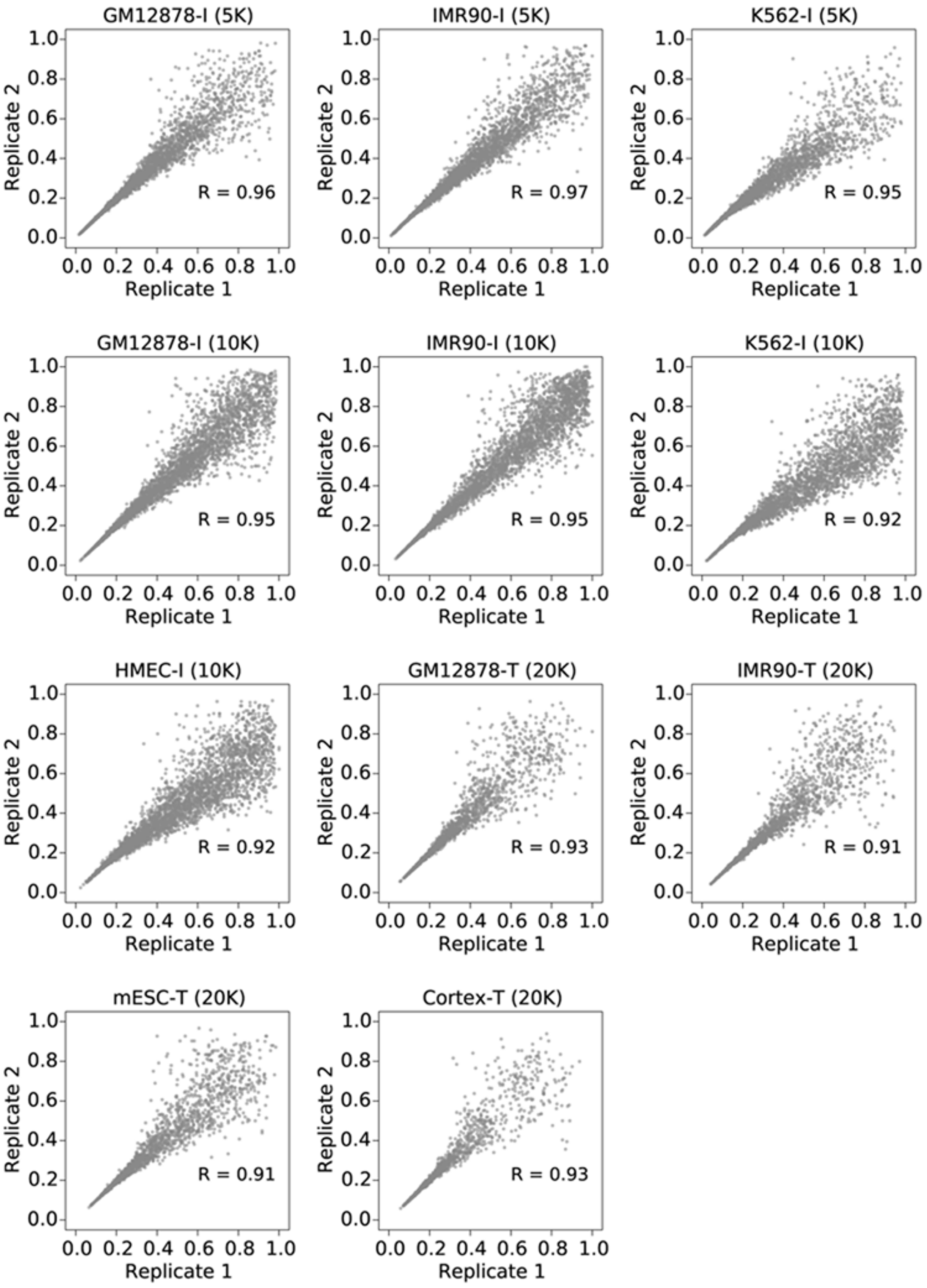

Supplementary figure 5. Pearson correlation between two replicates for defined parameter AP. The calculation from GM12878-I-R5 and GM12878-I-R7 is shown since these two replicates have highest and similar numbers of intra-chromosomal reads in GM12878-I (Supplementary table 1). Similarly, the correlation for IMR90-T is calculated from replicates IMR90-T-R1 and IMR90-T-R2 (Supplementary table 1).

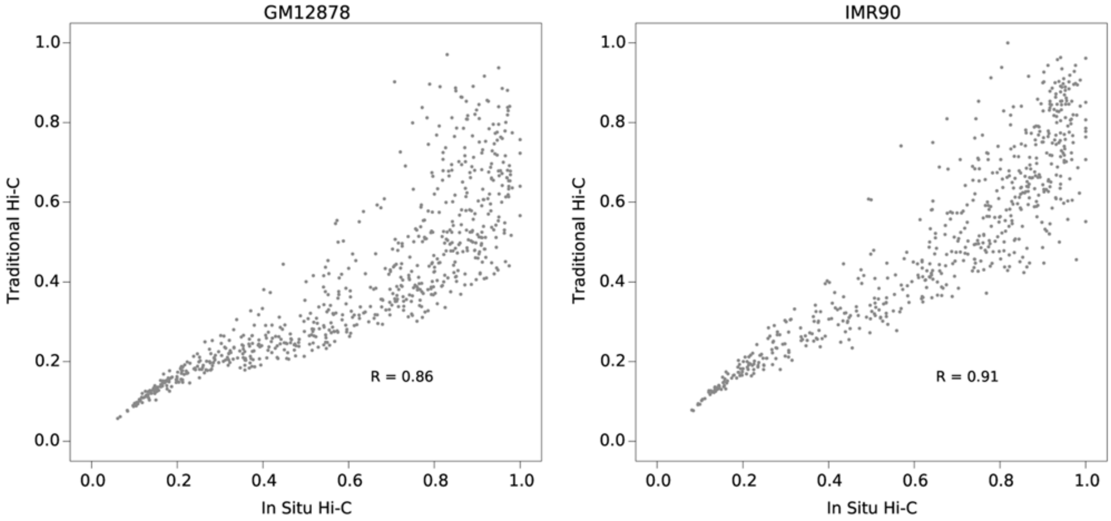

Supplementary figure 6. Pearson correlation between two experimental pipelines for defined parameter AP.

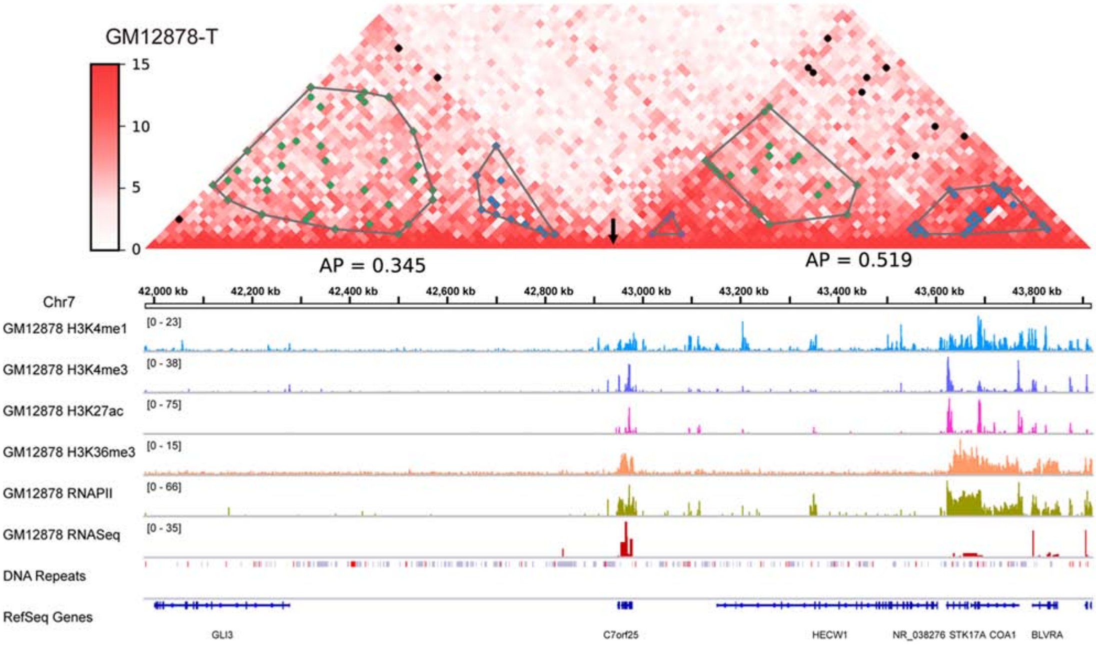

Supplementary figure 7. Structures and functions of two TADs from chromatin region (Chr7:41,980,000-43,920,000) in cell line GM12878-T at 20 kb resolution. The left TAD shows lower AP value and less active signals than the right TAD.

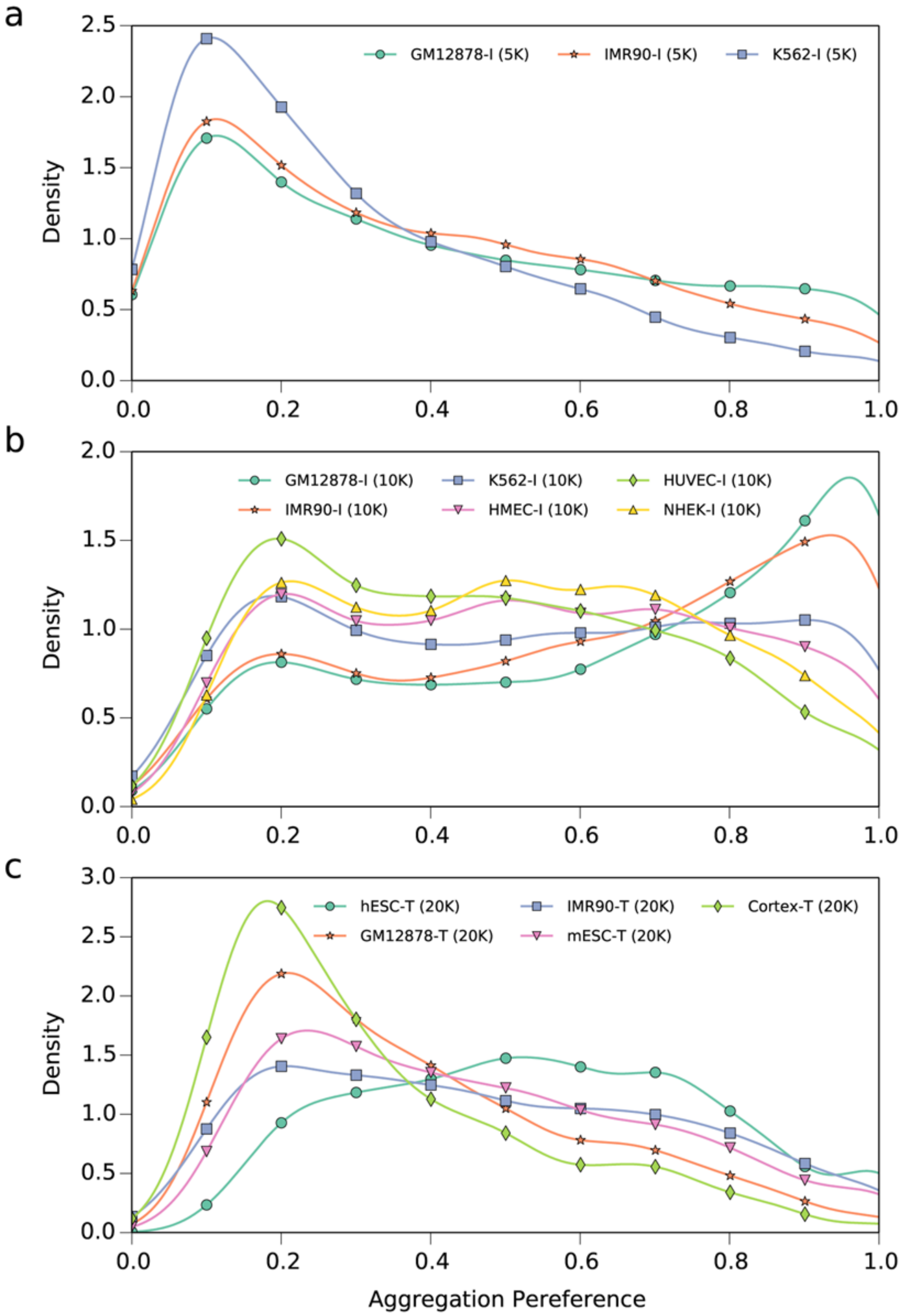

Supplementary figure 8. AP value distributions in different cases. (a) AP distributions of three deepest sequencing datasets at 5 kb resolution. (b) AP distributions of *in situ* Hi-C datasets at 10 kb resolution. (c) AP distributions of traditional Hi-C datasets at 20 kb resolution.

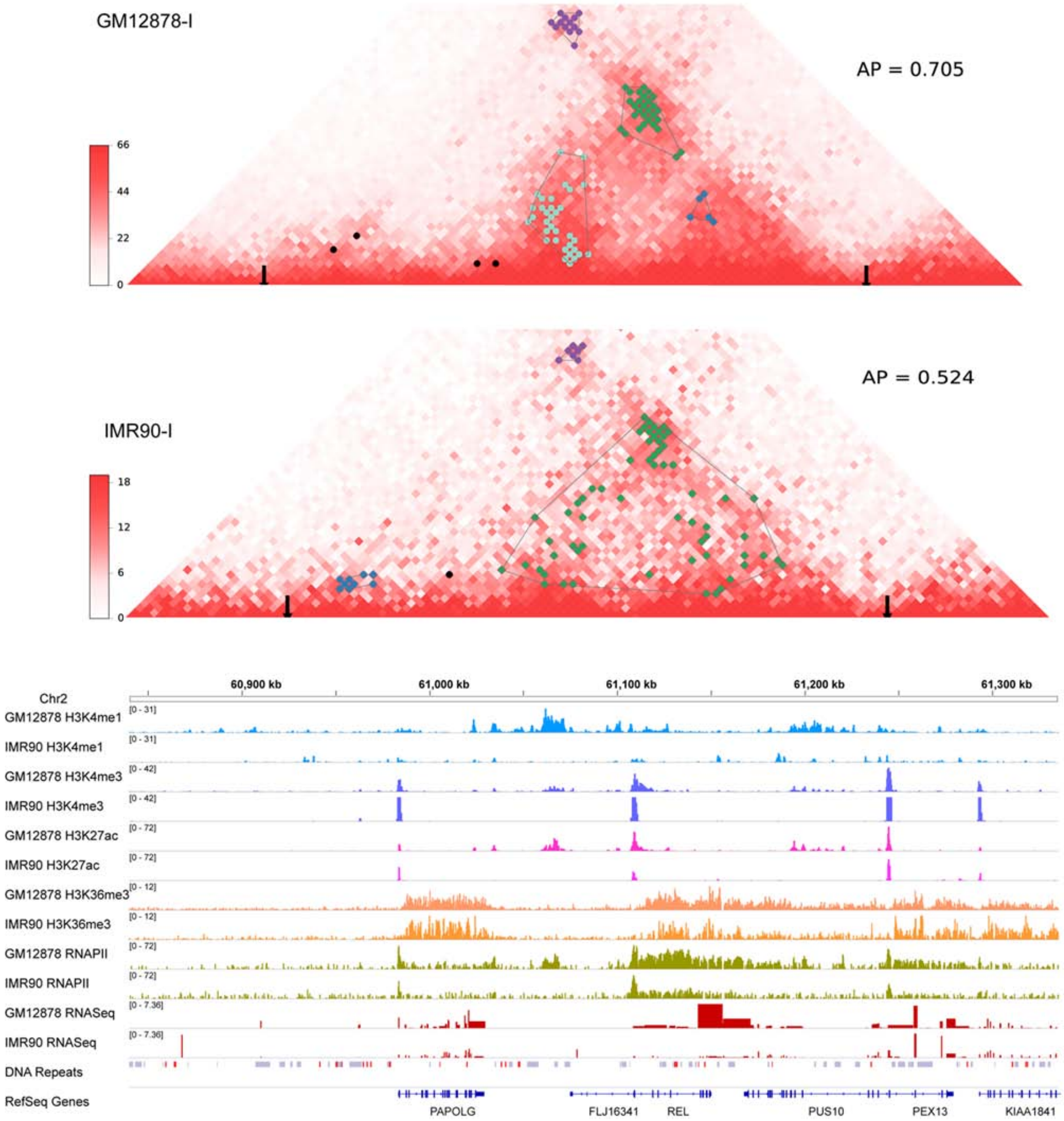

Supplementary figure 9. Block merging from cell lines GM12878-I to IMR90-I at 5 kb resolution. AP value decreases and the functional signals, especially enhancer signals (H3K4me1 and H3K2ac), become weak in IMR90-I. The TAD region and bidirectional extensions (Chr2: 60,840,000-61,335,000) are shown.

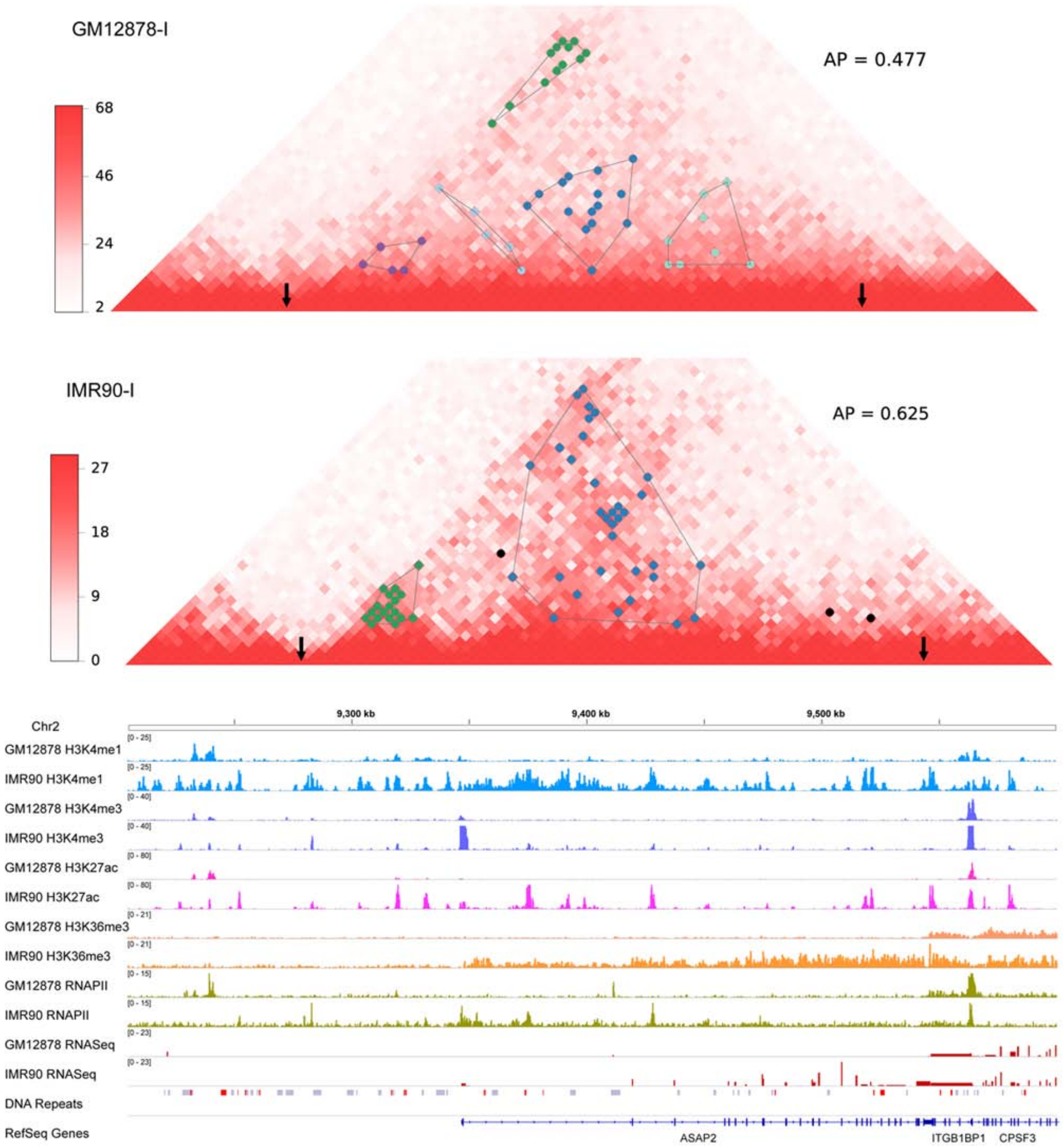

Supplementary figure 10. The complicated block change from cell lines GM12878-I to IMR90-I at 5 kb resolution. Block merging and disappearance concurrently occur in cell line IMR90-I. Besides, the left block is much strengthened in IMR90-I. AP value capture the overall structural and functional change. The TAD region and bidirectional extensions (Chr2: 9,205,000-9,600,000) are shown.

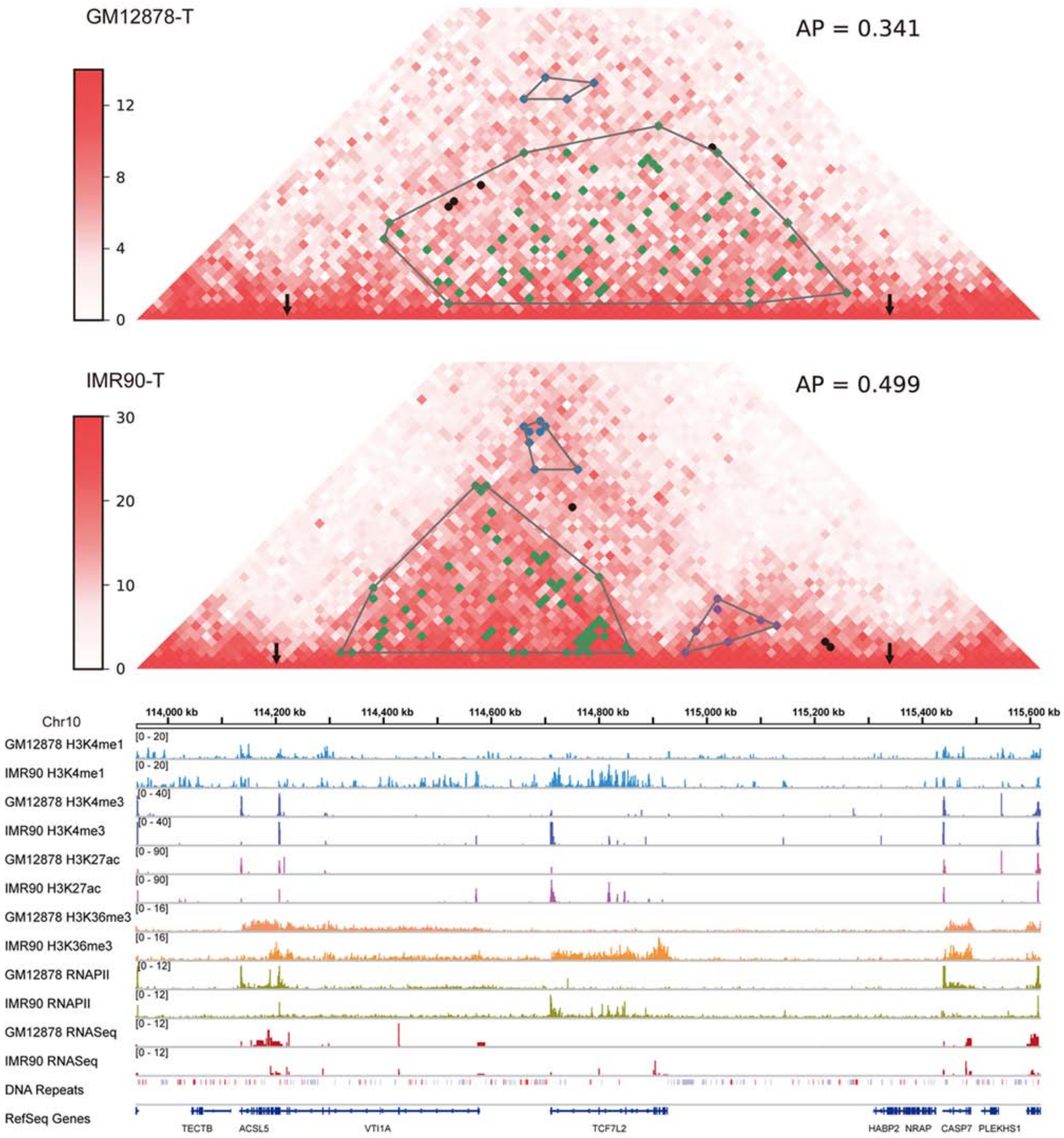

Supplementary figure 11. Block split from cell lines GM12878-T to IMR90-T at 20 kb resolution. AP value and active signals increase in IMR90-T. The TAD region and bidirectional extensions (Chr10: 113,940,000-115,620,000) are shown.

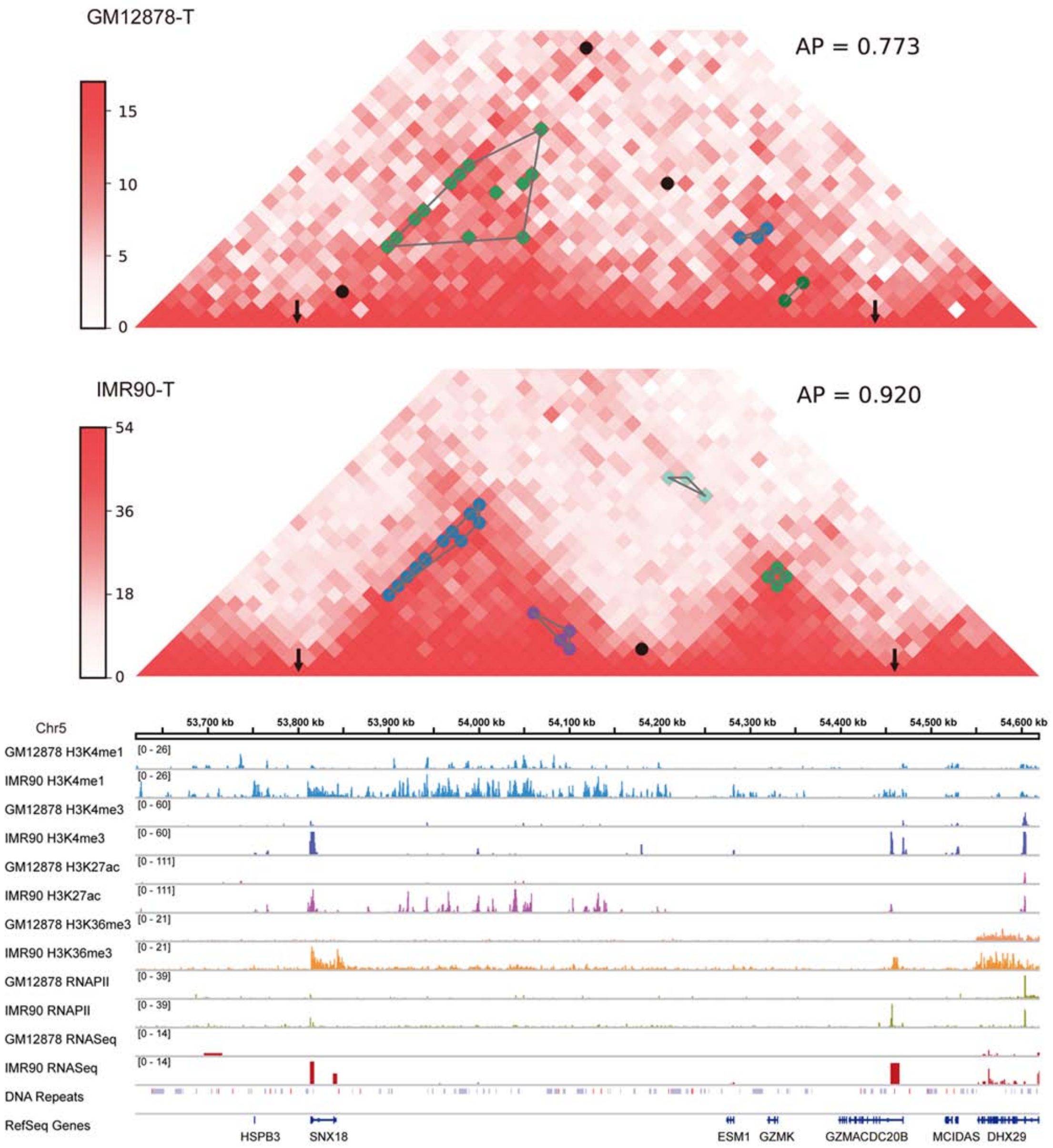

Supplementary figure 12. Concurrent block appearance and block change from cell lines GM12878-T to IMR90-T at 20 kb resolution. Though the structural change is complicated, AP value captures the overall structural and functional change in this TAD. The TAD region and bidirectional extensions (Chr2: 53,620,000-54,620,000) are shown.

Supplementary table 1. The summary of Hi-C data processing.

	Unique Mapped	After Filtering	Intra Chromosomal	Inter Chromosomal
GM12878-I-R1	3,348,688,198	1,469,688,054	1,102,045,856	367,642,198
GM12878-I-R2	296,330,900	125,091,645	97,672,320	27,419,325
GM12878-I-R3	359,550,346	136,398,536	104,101,010	32,297,526
GM12878-I-R4	166,748,340	78,454,464	59,147,824	19,306,640
GM12878-I-R5	611,717,683	247,884,358	196,786,619	51,097,739
GM12878-I-R6	102,976,917	45,760,527	33,651,053	12,109,474
GM12878-I-R7	660,326,459	336,751,148	226,549,236	110,201,912
GM12878-I-R8	306,430,686	147,015,617	111,055,822	35,959,795
GM12878-I-R9	220,851,001	108,846,198	74,111,336	34,734,862
IMR90-I-R1	840,668,928	407,216,087	338,804,700	68,411,387
IMR90-I-R2	572,292,610	226,978,449	192,453,064	34,525,385
K562-I-R1	959,481,811	375,354,045	303,083,165	72,270,880
K562-I-R2	293,624,676	142,683,547	93,992,431	48,691,116
HMEC-I-R1	408,353,405	170,440,720	125,780,802	44,659,918
HMEC-I-R2	74,580,713	26,698,914	20,961,482	5,737,432
HUVEC-I	649,267,201	266,679,439	186,675,650	80,003,789
NHEK-I	987,068,093	394,469,598	239,657,691	154,811,907
hESC-T-R1	202,644,947	13,400,964	7,889,451	5,511,513
hESC-T-R2	436,715,078	99,478,798	77,455,314	22,023,484
GM12878-T-R1	396,468,442	207,968,139	71,550,889	136,417,250
GM12878-T-R2	370,640,647	188,549,901	64,669,217	123,880,684
IMR90-T-R1	348,868,129	76,206,376	48,633,772	27,572,604
IMR90-T-R2	399,344,549	120,100,711	61,255,066	58,845,645
IMR90-T-R3	532,101,685	64,541,997	26,250,212	38,291,785
IMR90-T-R4	465,483,279	46,813,066	21,707,191	25,105,875
IMR90-T-R5	204,350,347	36,273,279	19,537,225	16,736,054
IMR90-T-R6	182,824,009	36,560,787	18,810,352	17,750,435
mESC-T-R1	408,136,131	103,523,149	84,773,361	18,749,788
mESC-T-R2	284,528,013	62,326,505	42,220,799	20,105,706
Cortex-T-R1	342,166,749	73,317,569	24,095,898	49,221,671
Cortex-T-R2	262,930,557	74,227,000	26,924,938	47,302,062

Supplementary table 2. Pearson correlation between AP change and block overlap on stable TADs across cell lines. The first column shows the two cell lines and corresponding resolution, and the second column shows the correlation coefficients. The number of stable TADs are shown in Supplementary figure 1.

	Pearson correlation coefficients
GM12878-I vs IMR90-I (5K)	-0.56
GM12878-I vs K562-I (5K)	-0.6
IMR90-I vs K562-I (5K)	-0.53
GM12878-I vs IMR90-I (10K)	-0.38
GM12878-I vs K562-I (10K)	-0.39
GM12878-I vs HMEC-I (10K)	-0.39
GM12878-I vs HUVEC-I (10K)	-0.51
GM12878-I vs NHEK-I (10K)	-0.45
IMR90-I vs K562-I (10K)	-0.37
IMR90-I vs HMEC-I (10K)	-0.41
IMR90-I vs HUVEC-I (10K)	-0.49
IMR90-I vs NHEK-I (10K)	-0.43
K562-I vs HMEC-I (10K)	-0.44
K562-I vs HUVEC-I (10K)	-0.53
K562-I vs NHEK-I (10K)	-0.47
HMEC-I vs HUVEC-I (10K)	-0.5
HMEC-I vs NHEK-I (10K)	-0.48
HUVEC-I vs NHEK-I (10K)	-0.47
hESC-T vs GM12878-T (20K)	-0.49
hESC-T vs IMR90-T (20K)	-0.48
GM12878-T vs IMR90-T (20K)	-0.56
mESC-T vs Cortex-T (20K)	-0.56
